# Supplementary material for: An image quality transfer approach for localising deep brain stimulation targets
Source: Imaging Neurosci (Camb). 2025 Nov 12;3:IMAG.a.1005. doi: 10.1162/IMAG.a.1005 (PMC12612099; doi:10.1162/IMAG.a.1005)
Supplement: Supplementary Material [file IMAG.a.1005_supp.pdf]

Supplemental Materials

SELECTION CRITERIA OF RELIABLE HQ-VIM

The subjects were split into two subsets, depending on the reliability of HQ-Vim. A subject's HQ-Vim has to pass 4 criteria in order to be accepted as "reliable":

1. the HQ-Vim's volume exceeds 20mm^3;
2. the HQ-Vim contains one blob;
3. Its correlation with the Vim from Thalamic DBS Connecitivity Atlas (Akram et al. 2018) is larger than 0.5;
4. Its center-of-mass is within 4mm of the center of mass of the Thalamic DBS Connecitivity Dentate Atlas;

The thresholds in 1., 3., and 4. are shown as vertical lines in Figure S1. 459 out of 1063 HCP subjects and 1445 out of 2760 UKB subjects passed all four criteria. These selection criteria exclude subjects whose HQ-Vim clusters lie too far away from the atlas to be considered as trustworthy, while preserving the inter-individual anatomical variability of the structure as much as possible. Note that, however, passing all the selection criteria does not necessarily guarantee the selected HQ-Vim as the perfect "gold standard". Instead, this only suggests that the HQ-Vim may serve as the gold standard Vim with relatively high confidence, as it is the best available estimate of the gold standard location of the Vim.

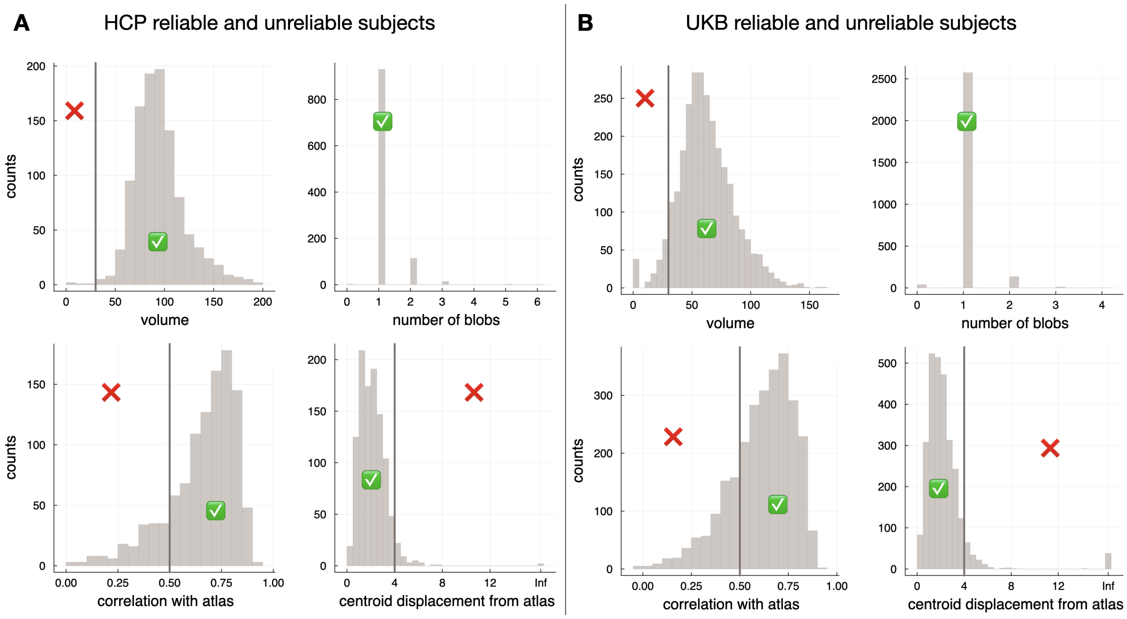

Figure S1. Split between the reliable and unreliable subsets. **(A)** Histograms of HQ-Vim's volume (mm^3), number of blobs, correlations and centroid displacement with the Thalamic DBS Connecitivity Atlas, for HCP subjects. **(B)** Equivalent plots to (A), for UKB subjects. Ticks indicate that the subjects pass the respective criteria, while crosses suggest their HQ-Vim were rejected as untrustworthy.

THE POWER OF THE POLYNOMIALS

This section describes how we chose the power of polynomials to expand the feature space $\mathbf{x}_i \in \mathbb{R}^{d\times 1}$. Specifically, $\phi(\mathbf{x}_i)$ undergoes polynomial feature expansion, augmented by a group-average term. Each feature x_k in $\mathbf{x}_i$ is transformed to include its corresponding polynomial terms $x_k^{p_1}, x_k^{p_2}, x_k^{p_3}$, along with the original feature values. These are then concatenated with g_i, the group-average Vim probability for the voxel, to form the expanded feature vector $\phi(\mathbf{x}_i) = [x_1, ...x_d, x_1^{p_1}, ...x_d^{p_1}, x_1^{p_2}, ...x_d^{p_2}, x_1^{p_3}, ...x_d^{p_3}, g_i]$. The powers of the polynomials $p_1 = 2, p_2 = 0.5, p_3 = 0.2$ were chosen by testing a range of power values on an independent subset (Figure S2), which appeared to be slightly better than the other choices.

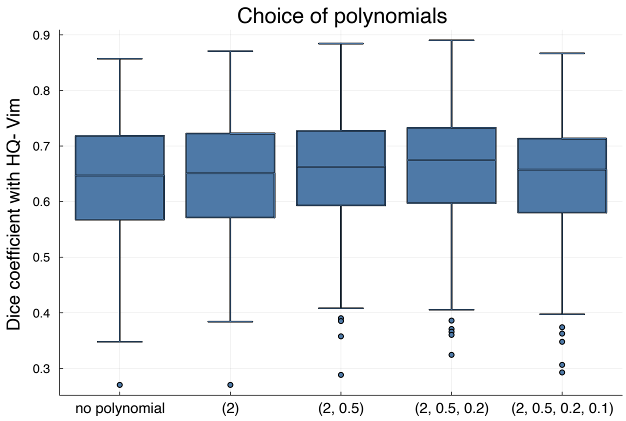

Figure S2. Choices of polynomial features. In addition to the original features (no polynomial), we tested a range of polynomial features on a smaller independent subset of subjects. The numbers in the x-axis denote the powers of polynomials used in the HQ-augmentation model, e.g., (2, 0.5, 0.2, 0.1) indicates that four additional polynomial features were included, with $p_1 = 2, p_2 = 0.5, p_3 = 0.2, p_4 = 0.1$ where p_1, p_2, p_3, p_4 were the powers. Overall, the choices of polynomials did not affect the Dice coefficient with HQ-Vim very much. However, (2, 0.5, 0.2), i.e., $p_1 = 2, p_2 = 0.5, p_3 = 0.2$, appeared to be slightly better than the other choices.

LIST OF ROIS

We provide a table listing the ROIs used in this study, as well as the respective tractography protocols and the procedures to obtain them.

ROI name (per hemisphere)	Procedures to obtain it
Thalamus	Obtained using T1, T2 and FA via FSL's MIST (Visser et al. 2016), a multimodal subcortical segmentation tool
Brainstem (both hemisphere)	Obtained via Freesurfer's subcortical segmentation tool, as released in the *aparc.a2009s.aseg.nii.gz files
Cerebellum grey matter (WM)	
Cerebellum grey matter (GM)	
Cerebrospinal fluid (CSF)	
Ventrical	
AR-1, AR-2, AR-3, AR-4, AR-5, and AR-6: six Acoustic Radiation fibre segments	Manually extracted from the Acoustic Radiation fibres (XTRACT atlas), and warped into individual space
STR-1, STR-2, STR-3, STR-4, STR-5, and STR-6: six Superior Thalamic Radiation WM segments	Manually extracted from the Superior Thalamic Radiation fibres (XTRACT atlas), and warped into individual space
OR-1, OR-2, OR-3, OR-4, OR-5, and OR-6: 6 Optic Radiation fibre segments	Manually extracted from the Optic Radiation fibres (XTRACT atlas), and warped into individual space
ATR-1, ATR-2, ATR-3, ATR-4, ATR-5, and ATR-6: 6 Anterior Thalamic Radiation fibre segments	Manually extracted from the Anterior Thalamic Radiation fibres (XTRACT atlas), and warped into individual space
FX-1, FX-2, FX-3, FX-4, and FX-5: five Fornix ROIs	Manually extracted from the Fornix (XTRACT atlas), and warped into individual space

SCPCT-brainstem, SCPCT-1, SCPCT-2, and SCPCT-3: four segments of the SCPCT tract, three lying between the brainstem ROI and ipsilateral thalamus ROI (SCPCT-1, SCPCT-2, SCPCT-3), one overlapping with the brainstem ROI (SCPCT-brainstem)	Extracted from the cerebellothalamic tract (Tang et al. 2018), and warped into individual space
4 white matter segments on the tracts that connect M1 and thalamus	Manually extracted from the tract that joins M1 and thalamus
4 white matter segments on the tracts that connect S1 and thalamus	Manually extracted from the tract that joins S1 and thalamus
G_and_S_frontomargin: Fronto-marginal gyrus (of Wernicke) and sulcus	Obtained from the Destrieux Atlas (Destrieux et al. 2010), via Freesurfer's cortical parcellation tool (https://surfer.nmr.mgh.harvard.edu/fswiki/CorticalParcellation) (Fischl et al. 2004; Desikan et al. 2006)
G_and_S_occipital_inf: inferior occipital gyrus (O3) and sulcus	
G_and_S_paracentral: Paracentral lobule and sulcus	
G_and_S_subcentral: Subcentral gyrus (central operculum) and sulci	
G_and_S_transv_frontopol: Transverse frontopolar gyri and sulci	
G_and_S_cingul-Mid-Ant: Middle-anterior part of the cingulate gyrus and sulcus	
G_and_S_cingul-Mid-Post: Middle-posterior part of the cingulate gyrus and sulcus	
G_cingul-Post-dorsal: Posterior-dorsal part of the cingulate gyrus	

G_cingul-Post-ventral: Posterior-ventral part of the cingulate gyrus
G_cuneus: Cuneus
G_front_inf-Opercular: Opercular part of the inferior frontal gyrus
G_front_inf-Orbital: Orbital part of the inferior frontal gyrus
G_front_inf-Triangul: Triangular part of the inferior frontal gyrus
G_front_middle: Middle frontal gyrus
G_front_sup: Superior frontal gyrus
G_Ins_lg_and_S_cent_ins: Long insular gyrus and central sulcus of the insula
G_insular_short : Short insular gyri
G_occipital_middle: Middle occipital gyrus
G_occipital_sup: Superior occipital gyrus
G_oc-temp_lat-fusifor: Lateral occipito-temporal gyrus
G_oc-temp_med-Lingual: Lingual gyrus, ligual part of the medial occipito-temporal gyrus
G_oc-temp_med-Parahip: Parahippocampal gyrus, parahippocampal part of the medial occipito-temporal gyrus
G_orbital: Orbital gyri
G_pariet_inf-Angular: Angular gyrus
G_pariet_inf-Supramar: Supramarginal gyrus
G_parietal_sup: Superior parietal lobule

G_postcentral: Postcentral gyrus
G_precentral: Precentral gyrus
G_precuneus: Precuneus
G_rectus: Straight gyrus
G_subcallosal: Subcallosal area, subcallosal gyrus
G_temp_sup-G_T_transv: Anterior transverse temporal gyrus
G_temp_sup-Lateral: Lateral aspect of the superior temporal gyrus
G_temp_sup-Plan_polar: Planum polare of the superior temporal gyrus
G_temp_sup-Plan_tempo : Planum temporale or temporal plane of the superior temporal gyrus
G_temporal_inf: Inferior temporal gyrus
G_temporal_middle: Middle temporal gyrus
Lat_Fis-ant-Horizont: Horizontal ramus of the anterior segment of the lateral sulcus
Lat_Fis-ant-Vertical: Vertical ramus of the anterior segment of the lateral sulcus
Lat_Fis-post: Posterior ramus (or segment) of the lateral sulcus
Medial_wall: Medial wall
Pole_occipital: Occipital pole
Pole_temporal: Temporal pole
S_calcarine: Calcarine sulcus
S_central: Central sulcus (Rolando's fissure)

S_cingul-Marginalis: Marginal branch (or part) of the cingulate sulcus
S_circular_insula_ant: Anterior segment of the circular sulcus of the insula
S_circular_insula_inf: Inferior segment of the circular sulcus of the insula
S_circular_insula_sup: Superior segment of the circular sulcus of the insula
S_collat_transv_ant: Anterior transverse collateral sulcus
S_collat_transv_post: Posterior transverse collateral sulcus
S_front_inf: Inferior frontal sulcus
S_front_middle: Middle frontal sulcus
S_front_sup: Superior frontal sulcus
S_interm_prim-Jensen: Sulcus intermedius primus
S_intrapariet_and_P_trans: Intraparietal sulcus (interparietal sulcus) and transverse parietal sulci
S_oc_middle_and_Lunatus: Middle occipital sulcus and lunatus sulcus
S_oc_sup_and_transversal: Superior occipital sulcus and transverse occipital sulcus
S_occipital_ant: Anterior occipital sulcus and preoccipital notch (temporo-occipital incisure)
S_oc-temp_lat: Lateral occipito-temporal sulcus

S_oc-temp_med_and_Lingual: Medial occipito-temporal sulcus (collateral sulcus) and lingual sulcus	
S_orbital_lateral: Lateral orbital sulcus	
S_orbital_med-olfact: Medial orbital sulcus (olfactory sulcus)	
S_orbital-H_Shaped: Orbital sulci (H-shaped sulci)	
S_parieto_occipital: Parieto-occipital sulcus (or fissure)	
S_pericallosal: Pericallosal sulcus (S of corpus callosum)	
S_postcentral: Postcentral sulcus	
S_precentral-inf-part: Inferior part of the precentral sulcus	
S_precentral-sup-part: Superior part of the precentral sulcus	
S_suborbital: Suborbital sulcus (sulcus rostrales, supraorbital sulcus)	
S_subparietal: Subparietal sulcus	
S_temporal_inf: Inferior temporal sulcus	
S_temporal_sup: Superior temporal sulcus	
S_temporal_transverse: Transverse temporal sulcus	

TABLE S1. The list of anatomical ROIs used in this study.

TRACTOGRAPHY PROTOCOLS

**Target mask (per hemisphere)**	**Waypoint mask**	**Exclusion mask**	**termination mask**
Contralateral cerebellar WM (dentate)	SCPCT-brainstem, contralateral cerebellar WM	Ipsilateral cerebellum WM and GM, CSF, Putamen, Caudate, contralateral thalamus	cortex
SCPCT-brainstem (the segment of SCPCT that overlap with the brainstem ROI)	SCPCT-brainstem	Ipsilateral cerebellum WM and GM, CSF, Putamen, Caudate, contralateral thalamus	cortex
SCPCT-1, SCPCT-2, and SCPCT-3	Same as the respective target mask	Ipsilateral cerebellum WM and GM, CSF, Putamen, Caudate, contralateral thalamus	SCPCT-brainstem, cortex
G_and_S_frontomargin			
G_and_S_occipital_inf			
G_and_S_paracentral			
G_and_S_subcentral			
G_and_S_transv_frontopol			
G_and_S_cingul-Mid-Ant			
G_and_S_cingul-Mid-Post			
G_cingul-Post-dorsal			
G_cingul-Post-ventral			
G_cuneus	same as target mask	Ipsilateral cerebellum, CSF	cortex

G_front_inf-Opercular			
G_front_inf-Orbital			
G_front_inf-Triangul			
G_front_middle			
G_front_sup			
G_Ins_lg_and_S_cent_ins			
G_insular_short			
G_occipital_middle			
G_occipital_sup			
G_oc-temp_lat-fusifor			
G_oc-temp_med-Lingual			
G_oc-temp_med-Parahip			
G_orbital			
G_pariet_inf-Angular			
G_pariet_inf-Supramar			
G_parietal_sup			
G_postcentral			
G_precentral (M1)			
G_precuneus			
G_rectus			
G_subcallosal			
G_temp_sup-G_T_transv			
G_temp_sup-Lateral			
G_temp_sup-Plan_polar			
G_temp_sup-Plan_tempo			
G_temporal_inf			
G_temporal_middle			

Lat_Fis-ant-Horizont			
Lat_Fis-ant-Vertical			
Lat_Fis-post			
Medial_wall			
Pole_occipital			
Pole_temporal			
S_calcarine			
S_central			
S_cingul-Marginalis			
S_circular_insula_ant			
S_circular_insula_inf			
S_circular_insula_sup			
S_collat_transv_ant			
S_collat_transv_post			
S_front_inf			
S_front_middle			
S_front_sup			
S_interm_prim-Jensen			
S_intrapariet_and_P_trans			
S_oc_middle_and_Lunatus			
S_oc_sup_and_transversal			
S_occipital_ant			
S_oc-temp_lat			
S_oc-temp_med_and_Lingual			
S_orbital_lateral			
S_orbital_med-olfact			
S_orbital-H_Shaped			

S_parieto_occipital			
S_pericallosal			
S_postcentral			
S_precentral-inf-part			
S_precentral-sup-part			
S_suborbital			
S_subparietal			
S_temporal_inf			
S_temporal_sup			
S_temporal_transverse			
AR1-AR6			
STR1-STR6			
ATR1-ATR6			
OR1-OR6			
FX1-FX5			
S1-1-4			
M1-1-4			

TABLE S2. The list of target, waypoint, exclusion and termination masks used in tractography.

MEAN-FIELD APPROXIMATION OF THE CRF DISTRIBUTION

As mentioned in section 2, we seek to maximise the probability of reproducing the exact same HQ-Vim label assignment $\mathbf{y}$ on its low-quality counterparts by minimising the cross entropy in Equation (1). Due to the inter-dependency of neighbouring voxels, the exact analytical minimisation is intractable. Thus, we approximate the CRF distribution $P(\mathbf{y}|\mathbf{X})$ by a simpler function $Q(\mathbf{y})$, and iteratively solve this optimisation problem, as explained below.

Suppose we have K classes (here $K = 2$). To initialise the model, we derived the initial coefficients $\hat{\mathbf{W}}^{(0)} = [\hat{\mathbf{w}}_1^{(0)}, ...\hat{\mathbf{w}}_K^{(0)}]$ by optimising the following (without regularisation terms):

$$\hat{\mathbf{W}}^{(0)} = \arg\max_{\mathbf{W}} \sum_i^V \sum_j^K t_{ij} \log(P'(y_i|\mathbf{x}_i)) \tag{S1}$$

where $P'(y_i|\mathbf{x}_i)$ is the likelihood without considering local smoothness of the label assignment, i.e., ignoring the pairwise loss term in Equation (3):

$$P'(y_i = l|\mathbf{x}_i) = \frac{\exp(-\psi_u(y_i = l|\mathbf{x}_i))}{\sum_k^K \exp(-\psi_u(y_i = k|\mathbf{x}_i))} \tag{S2}$$

where, as defined before, $\psi_u(y_i = l|\mathbf{x}_i) = \mathbf{w}_l^T \phi(\mathbf{x}_i)$. The coefficients $\hat{\mathbf{W}}^{(0)}$ were used to initialise $Q(\mathbf{y})$ using Equation (S2), i.e., $Q_i(y_i = l) \leftarrow P'(y_i = l|\mathbf{x}_i)$ evaluated at $\hat{\mathbf{W}}^{(0)}$.

After initialisation, $Q_i(y_i = l)$ is updated as the weighted sum of its neighbouring Q values, $\tilde{Q}_i(y_i = l) \leftarrow \rho \sum_{j \in \mathcal{N}_i} k(\phi(\mathbf{x}_i), \phi(\mathbf{x}_j)) Q_j(y_j = l)$, where $\tilde{Q}_i(y_i = l)$ is the updated Q value. This is the message-passing step. Next, label incompatibility was calculated as a penalty to encourage local smoothness. The incompatibility for label l at a given voxel i, denoted by $\hat{Q}_i(y_i = l)$, was calculated as the sum of the updated $\tilde{Q}_i$ that takes a different label, i.e., $\hat{Q}_i(y_i = l) \leftarrow \sum_{l'} \mu(l, l') \tilde{Q}_i(y_i = l')$. Next, this penalty incurred by incompatibility was subtracted from the unary inputs $\psi_u(y_i = l|\mathbf{x}_i)$, i.e., $Q_i(y_i = l) \leftarrow \frac{1}{Z_i'} \exp(-\psi_u(y_i = l|\mathbf{x}_i) - \hat{Q}_i(y_i = l))$, where $Z_i' = \sum_k^K \exp(-\psi_u(y_i = k|\mathbf{x}_i) - \hat{Q}_i(y_i = k))$ is the normalisation constant. The above steps were repeated until Q converges. The resulting $Q_i(y_i|\mathbf{x}_i)$ is an approximation of the likelihood $P(y_i|\mathbf{x}_i)$, and was used when calculating the cross entropy in Equation (1). This cross entropy (1) was

minimised in a mini-batch style via an ADAM optimiser (Kingma and Ba 2014) with learning rate 0.01, in which the connectivity features $\mathbf{X}$ of each subject served as a mini-batch. The model was trained using only the reliable subjects. To clarify, "reliable" refers to the subjects that passed the four selection criteria. These reliable subjects were further divided into two separate groups or "folds". The model was trained using one fold at a time, with the other fold and "unreliable" subjects (those who did not pass the quality control) used for testing. This approach ensured that our model was exposed to and learned from the most accurate data available.

The pseudo code of the above steps is summarised in Table S3.

**Algorithm: Mean-field iteration in CRF**
1: $Q_i(y_i = l) \leftarrow \exp(-\psi_u(y_i = l\|\mathbf{x}_i))/\sum_k \exp(-\psi_u(y_i = k\|\mathbf{x}_i))$ *# Initialization*
2: **while** not converged **do**
3: $\tilde{Q}_i(y_i = l) \leftarrow \rho \sum_{j \in \mathcal{N}_i} k(\phi(\mathbf{x}_i), \phi(\mathbf{x}_j)) Q_j(y_j = l)$ *# Message Passing*
4: $\hat{Q}_i(y_i = l) \leftarrow \sum_{l'} \mu(l, l') \tilde{Q}_i(y_i = l')$ *# Compatibility with neighbours*
5: $Q_i(y_i = l) \leftarrow \frac{1}{Z_i'} \exp(-\psi_u(y_i = l\|\mathbf{x}_i) - \hat{Q}_i(y_i = l))$ *# Approximate $P(y_i = l\|\mathbf{x}_i)$*
6: **end while**

TABLE S3. Pseudo code of the mean-field iteration to solve $Q(\mathbf{y})$, an approximation of the CRF distribution $P(\mathbf{y}|\mathbf{X})$.

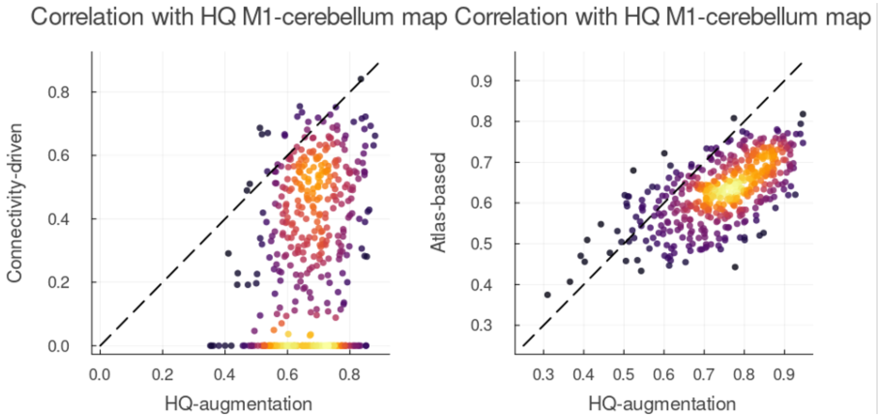

Figure S3. Threshold-free validation using correlation with HQ M1×cerebellum maps on LQ-LowAngular dataset. Pearson's correlation between continuous predicted probability maps and high-quality M1×cerebellum tract density product serves as an additional validation metric without thresholding. Results confirm the main findings from Dice coefficient and centroid displacement analyses: HQ-augmentation achieves the highest correlation with the HQ reference maps, followed by atlas-based and connectivity-driven approaches.

	LQ-LowAngular		*LQ-LowSpatial*	
	Dice coefficient	Centroid displacement	Dice coefficient	Centroid displacement
HQ-augmentation vs connectivity-driven	<1e-99	<1e-54	<1e-99	<1e-89
HQ-augmentation vs atlas-based	<1e-10	<1e-17	0.0043	<1e-17
	LQ-LowAngular-LowSpatial		LQ-UKB	
	Dice coefficient	Centroid displacement	Dice coefficient	Centroid displacement
HQ-augmentation vs connectivity-driven	<1e-72	<1e-38	<1e-96	<1e-66
HQ-augmentation vs atlas-based	<1e-4	1e-12	<1e-19	<1e-12

TABLE S4. Statistical comparison of HQ-augmentation versus alternative methods. Two-sided paired t-tests were conducted to compare HQ-augmentation performance against connectivity-driven and atlas-based approaches across four low-quality datasets (LQ-LowAngular, LQ-LowSpatial, LQ-LowAngular-LowSpatial, and LQ-UKB). P-values were Bonferroni corrected for multiple comparisons (16 tests total: 2 methods × 4 datasets × 2 metrics).

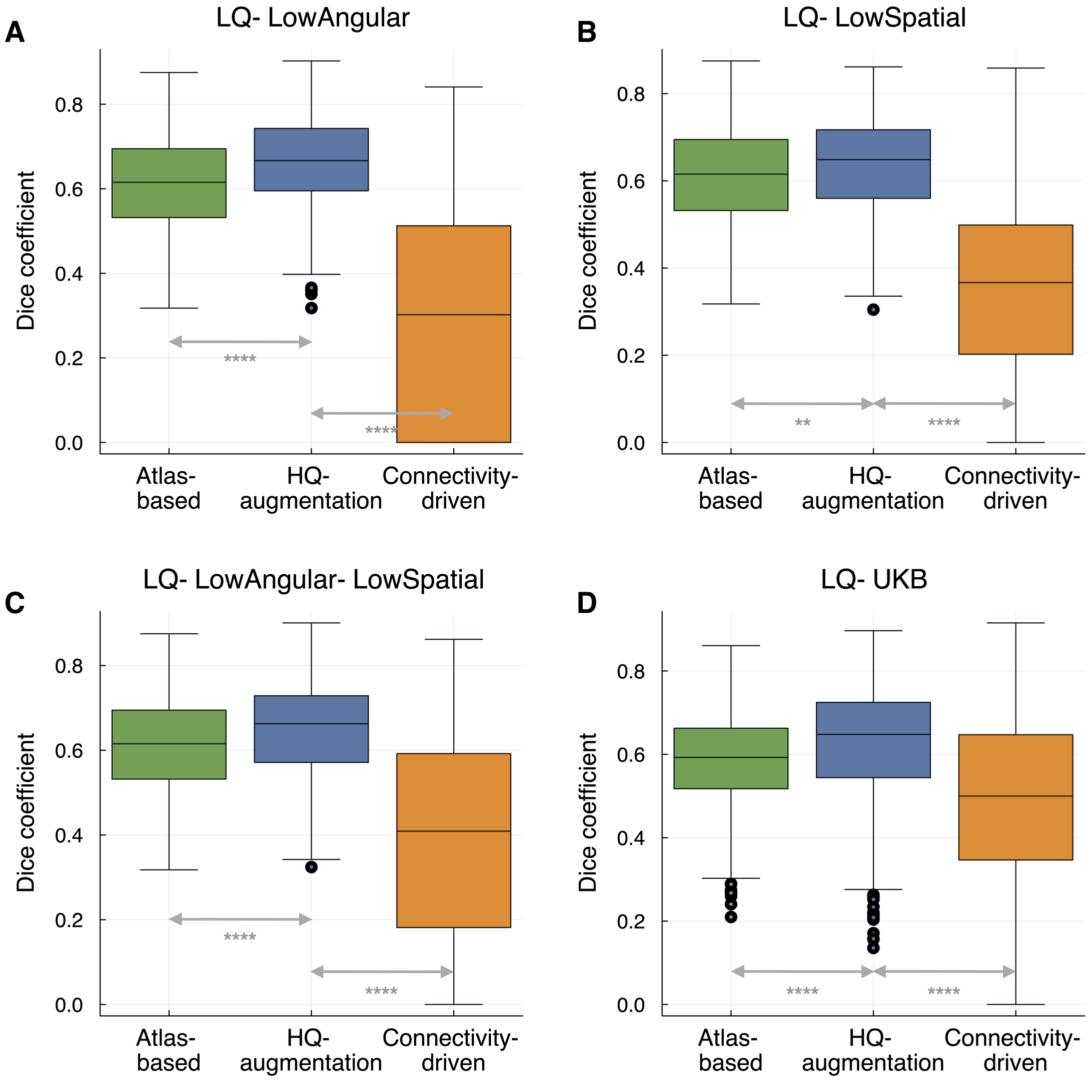

Figure S4. Boxplots of the Dice coefficient with HQ-Vim on surrogate low-quality datasets. (**A**). Dice coefficient with the HQ-Vim, produced by the atlas-based (green), HQ-augmentation (blue), and connectivity-driven (orange) approach, on *LQ-LowAngular*. (**B**). Equivalent plots of (**A**), on *LQ-LowSpatial*. (**C**). Equivalent plots of (**A**), on *LQ-LowAngular-LowSpatial*. (**D**). Equivalent plots of (**A**), on LQ-UKB (where the HQ-augmentation model was trained on HCP and applied on LQ-UKB). Please see TABLE S4 for the statistical tests.

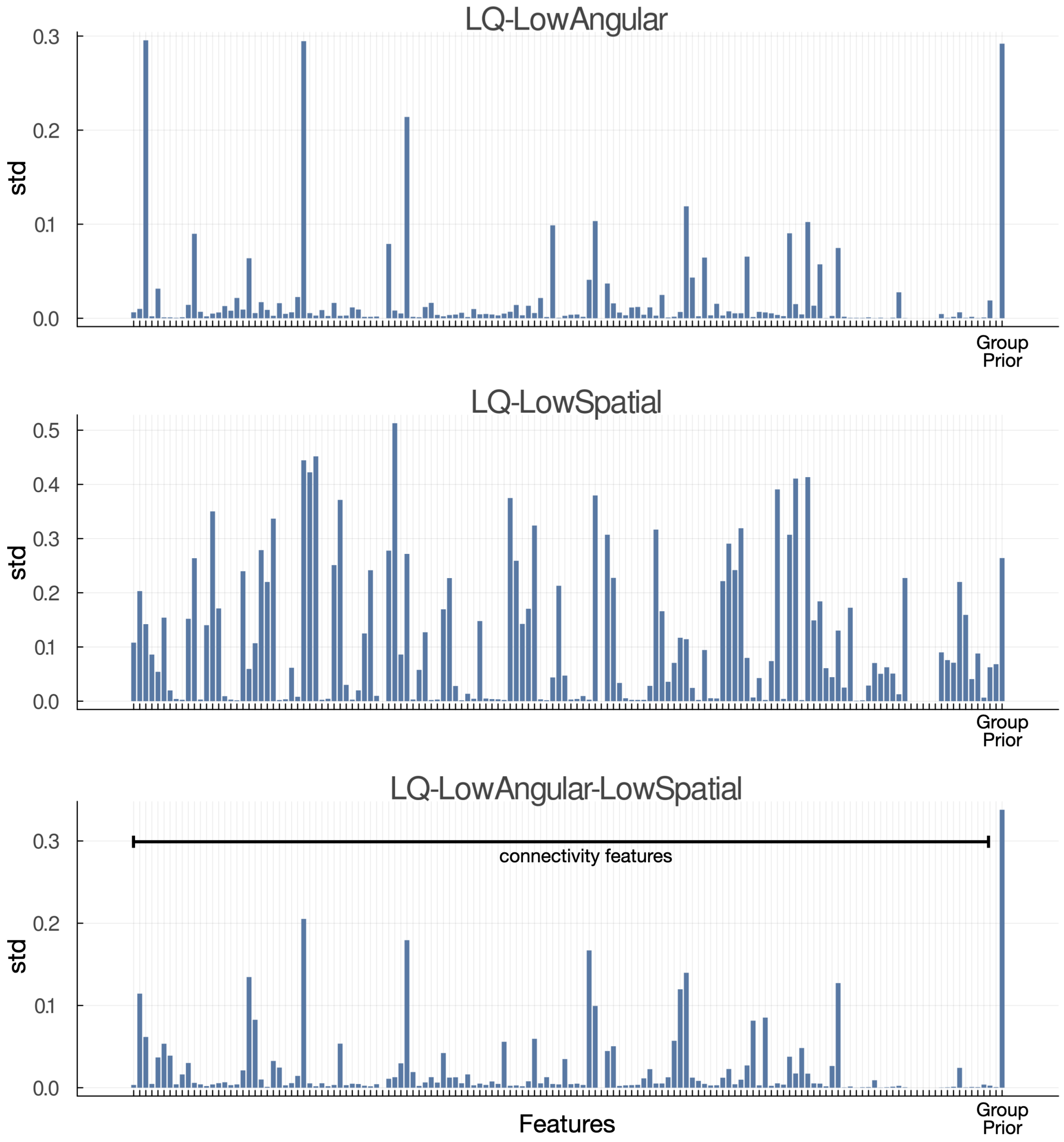

Figure S5. Relative contributions of the features in HQ-augmentation model for *LQ-LowAngular*, *LQ-LowSpatial*, *LQ-LowAngular-LowSpatial*. We plotted the std. of each feature contribution (i.e., the feature times its coefficient) across 50 subjects. The group-average prior feature contributed the most to Vim prediction, which is unsurprising given that the gold standard HQ-Vim labels were confined to be close to the atlas-defined Vim. For the purpose of visualisation, we summed the std. across the polynomials of a given connectivity feature. For example, the std. for M1 shown here was obtained by summing the std. of M1's polynomial features. Specifically, suppose β_1 is the coefficient for $\mathbf{x}_{\mathrm{M1}}$, and β_2 for $\mathbf{x}^2_{\mathrm{M1}}$, β_3 for $\mathbf{x}^{0.5}_{\mathrm{M1}}$, and β_4 for $\mathbf{x}^{0.2}_{\mathrm{M1}}$; then the std for M1 shown here is $std(\beta_1\mathbf{x}_{\mathrm{M1}}) + std(\beta_2\mathbf{x}^2_{\mathrm{M1}}) + std(\beta_3\mathbf{x}^{0.5}_{\mathrm{M1}}) + std(\beta_4\mathbf{x}^{0.2}_{\mathrm{M1}})$ across 50 subjects.

References

Akram, H., Dayal, V., Mahlknecht, P., Georgiev, D., Hyam, J., Foltynie, T., Limousin, P., De Vita, E., Jahanshahi, M., Ashburner, J., Behrens, T., Hariz, M., and Zrinzo, L. (2018). “Connectivity derived thalamic segmentation in deep brain stimulation for tremor.” *NeuroImage: Clinical*, 18, 130–142.

Desikan, R. S., Ségonne, F., Fischl, B., Quinn, B. T., Dickerson, B. C., Blacker, D., Buckner, R. L., Dale, A. M., Maguire, R. P., Hyman, B. T., Albert, M. S., and Killiany, R. J. (2006). “An automated labeling system for subdividing the human cerebral cortex on mri scans into gyral based regions of interest.” *Neuroimage*, 31(3), 968–980.

Destrieux, C., Fischl, B., Dale, A., and Halgren, E. (2010). “Automatic parcellation of human cortical gyri and sulci using standard anatomical nomenclature.” *Neuroimage*, 53(1), 1–15.

Fischl, B., Van Der Kouwe, A., Destrieux, C., Halgren, E., Ségonne, F., Salat, D. H., Busa, E., Seidman, L. J., Goldstein, J., Kennedy, D., Caviness, V., Makris, N., Rosen, B., and Dale, A. M. (2004). “Automatically parcellating the human cerebral cortex.” *Cerebral cortex*, 14(1), 11–22.

Kingma, D. P. and Ba, J. (2014). “Adam: A method for stochastic optimization.” *arXiv preprint arXiv:1412.6980*.

Tang, Y., Sun, W., Toga, A. W., Ringman, J. M., and Shi, Y. (2018). “A probabilistic atlas of human brainstem pathways based on connectome imaging data.” *Neuroimage*, 169, 227–239.

Visser, E., Keuken, M. C., Douaud, G., Gaura, V., Bachoud-Levi, A.-C., Remy, P., Forstmann, B. U., and Jenkinson, M. (2016). “Automatic segmentation of the striatum and globus pallidus using mist: Multimodal image segmentation tool.” *NeuroImage*, 125, 479–497.
